# Active chitosan coating to inhibit decay and maintain mango postharvest quality

**DOI:** 10.1002/jsfa.70256

**Published:** 2025-10-21

**Authors:** Angelucia Gonçalves Parente, Ana Caroliny de Souza, Henrique Sátiro Gama e Silva, Danillo Sales Rosa, Mateus Matiuzzi da Costa, Bruna Parente de Carvalho Pires, Pedro Martins Ribeiro Júnior, Sérgio Tonetto de Freitas, David Fernando de Morais Neri

**Affiliations:** ^1^ Institute of Materials Science, Universidade Federal do Vale do São Francisco Juazeiro Brazil; ^2^ Universidade Federal Rural de Pernambuco Recife Brazil; ^3^ Universidade Federal do Vale do São Francisco Petrolina Brazil; ^4^ Brazilian Agricultural Research Corporation, Tropical Semi‐Arid Embrapa Petrolina Brazil

**Keywords:** biodegradable, antifungal, stability, preservation, nanotechnology

## Abstract

**BACKGROUND:**

Microbial decay limits the storage, quality and trade of mangoes (*Mangifera indica*). We developed an active chitosan coating containing zinc oxide nanoparticles (ZnO‐NPs) and *Eucalyptus radiata* essential oil (EEO) to inhibit decay and preserve postharvest quality of mango. Chitosan (cationic) can disrupt microbial membranes; ZnO‐NPs act mainly via reactive‐oxygen‐species generation and Zn^2+^ interactions; EEO (rich in oxygenated monoterpenes) perturbs lipid bilayers, offering complementary antimicrobial actions.

**RESULTS:**

*In vitro*, the developed coating achieved 97.28% inhibition of *Lasiodiplodia theobromae* mycelial growth (plate assay) and yielded the lowest MIC/MBC values against *Staphylococcus aureus* and *Escherichia coli*. *In vivo*, for ‘Tommy Atkins’ mangoes stored at 9 °C for 28 days, coated fruit retained higher firmness (7.30 N *versus* 3.42 N, *P* ≤ 0.05), showed smaller skin colour changes and exhibited a 7‐day delay in the respiratory peak. Postharvest decay severity in coated fruit was reduced by *ca* 64.3%, compared to uncoated fruit. The developed coating resulted in the lowest AUDPC (17.9) against anthracnose.

**CONCLUSION:**

The synergistic action of chitosan, ZnO‐NPs and EEO enhanced antimicrobial efficacy and preserved postharvest quality, reducing decay and the need for synthetic fungicide application on mango. The coating can be integrated after sanitisation and before packing, and is compatible with cold‐chain logistics at 9 °C. These outcomes support longer distribution windows and improved market access for the mango industry. © 2025 The Author(s). *Journal of the Science of Food and Agriculture* published by John Wiley & Sons Ltd on behalf of Society of Chemical Industry.

## INTRODUCTION

The use of edible coatings has gained prominence as a sustainable and effective strategy for preserving postharvest quality, particularly in tropical fruits such as mangoes (*Mangifera indica*).^1^ Coatings help protect fruit from physical, chemical and microbial deterioration, thereby extending postharvest life, improving marketability and preserving nutritional and nutraceutical properties.^2^, ^3^ Mangoes, known for their high perishability, hold significant economic and nutritional relevance in many regions worldwide.^4^, ^5^ However, their rapid postharvest deterioration presents a major challenge, emphasising the need for effective preservation techniques.^6^ Postharvest losses directly affect profitability and the reliability of supply in the global mango market, reinforcing the need for effective, scalable preservation strategies.^5^

Among emerging strategies, edible coatings stand out due to their multifunctionality. When applied to fruit surfaces, coatings act as semipermeable barriers that reduce moisture loss, limit respiration rates and inhibit microbial growth, often outperforming other conservation technologies.^7^ In particular, chitosan (CS)‐based coatings have been widely studied due to the polymer's natural antimicrobial, biodegradable and film‐forming properties.^8^ Briefly, CS (a cationic polysaccharide) can interact with and disrupt microbial cell envelopes^8^; zinc oxide nanoparticles (ZnO‐NPs) exert antimicrobial action mainly via generation of reactive oxygen species (ROS) and Zn^2+^ interactions at cell surfaces^9^; and *Eucalyptus radiata* essential oil (EEO), rich in oxygenated monoterpenes, can disrupt lipid bilayers and membrane‐associated functions.^10^ The incorporation of such functional additives into CS has been reported to enhance both antimicrobial and antioxidant performance.^8^, ^9^, ^10^

Despite progress in this field, limited work has addressed the application of this specific multicomponent system to mangoes. The combination of CS, ZnO‐NPs and EEO represents a promising yet underexplored approach to reduce decay incidence and maintain postharvest quality of mango, with potential benefits for supply chains and market access.^11^ Here, we address this gap by evaluating the effect of a multicomponent CS coating co‐formulated with ZnO‐NPs and EEO on maintaining postharvest quality and reducing decay incidence in ‘Tommy Atkins’ mangoes during cold storage.

Accordingly, the objectives of the study reported here were to develop and evaluate the effect of an active CS‐based coating, enriched with ZnO‐NPs and EEO, on inhibiting decay incidence and maintaining the postharvest quality of ‘Tommy Atkins’ mangoes during storage.

## MATERIALS AND METHODS

The experiments were conducted at the Laboratory Block of the Universidade Federal do Vale do São Francisco (UNIVASF) and at the Postharvest and Phytopathology Laboratories of Tropical Semi‐arid Embrapa, both located in Petrolina, Pernambuco, Brazil.

### Materials

Chitosan from shrimp shells (degree of deacetylation ≥ 75%), Tween 80 (Polysorbate 80), glycerol and a ZnO‐NP dispersion were purchased from Sigma‐Aldrich (St Louis, MO, USA). The ZnO dispersion contained 20 wt% ZnO in water; supplier specifications indicated nominal primary particle size < 100 nm (TEM), average particle size ≤ 40 nm (APS) and specific surface area ≥ 40 m^2^ g^−1^. EEO was obtained from doTERRA® (batch no. 2232627BR); according to the supplier, its major constituents were 1,8‐cineole (63.93%), *α*‐terpineol (14.30%), limonene (4.72%) and *α*‐pinene (2.16%). The fungal strains *Colletotrichum* sp. and *Lasiodiplodia theobromae* were isolated from naturally infected mangoes. The bacterial strains *Staphylococcus aureus* (ATCC 25923) and *Escherichia coli* (ATCC 25922) were obtained from a reference collection. Glacial acetic acid and all other reagents were of analytical grade and used as received.

### Preparation of coating solution

The CS‐based coatings were prepared according to Hafsa *et al*.,^12^ with slight modifications to incorporate the selected active agents (addition of a plasticiser/emulsifier and the actives at the stated concentrations). The coating solution was obtained by dissolving 1.0% (w/v) CS in 1.0% (v/v) glacial acetic acid under constant stirring at 50 ± 5 °C for 60 min, followed by filtration to remove insoluble particles.

For formulations containing a plasticiser and emulsifier, glycerol (1.0% v/v) and Tween 80 (0.5% v/v) were added, and the mixture was stirred at room temperature for 30 min. Functionalised solutions were prepared by incorporating EEO (1.0% v/v) and a ZnO‐NP stock dispersion at 1.0% (v/v), yielding a final ZnO content of *ca* 2 mg mL^−1^ (solids basis, from the 20 wt% stock reported in the previous section). All additions were made under continuous stirring at room temperature for 60 min.

Qualitatively, the final coating solution was homogeneous, slightly yellow (as expected for CS solutions) and sufficiently viscous for manual spreading; no rheological measurements were performed. The final composition of the active CS coating was used consistently across all *in vitro* and *in vivo* assays. The composite formulation (T2; see Table 1) was used unchanged across the *in vitro* and *in vivo* assays.

**Table 1:** Summary of treatments and formulations (T1–T8) for ‘Tommy Atkins’ mangoes: component composition and inoculation status across assays (T1–T7 inoculated with *Colletotrichum* sp.; T8 non‐inoculated, negative control)

Sample	Treatment	Description	Fungal inoculum
T1	Chitosan 1%^a^	1% chitosan	Yes
T2	Composite formulation (test)^a^	1% chitosan + 1% glycerol + 0.25% Tween 80 + 0.5% ZnO‐NPs + 0.5% eucalyptus essential oil	Yes
T3	Tween 80	0.25% Tween 80 in water	Yes
T4	Acetic acid	1% acetic acid	Yes
T5	ZnO‐NPs	0.5% zinc oxide nanoparticles in water	Yes
T6	Tween 80 + EEO	0.25% Tween 80 + 0.5% eucalyptus essential oil in water	Yes
T7	Positive control	Sterile distilled water	Yes
T8	Negative control	Sterile distilled water	No

^a^ Prepared in 1% acetic acid.

### Direct *in vitro* effect of formulation against microorganisms

#### *In vitro* antibacterial activity against *S. aureus* and *E. coli*

The minimum inhibitory concentration (MIC; the lowest concentration capable of preventing visible growth in broth) and the minimum bactericidal concentration (MBC; the lowest concentration yielding no colonies on drug‐free agar after subculture) were determined by the broth microdilution method, as described by Franciscato *et al*.,^13^ with minor modifications. Methicillin‐sensitive *Staphylococcus aureus* (ATCC 25923) and *Escherichia coli* (ATCC 25922) were employed.

The assays were performed in 96‐well microplates containing Mueller–Hinton broth (MHB). To evaluate the individual contribution of each constituent and their combinations to the antimicrobial activity, different experimental samples were prepared, comprising: (i) chitosan (CS) 1% w/v; (ii) CS 1% w/v + glycerol (Gly) 1% v/v; (iii) Gly 1% v/v + Tween 80 (T80) 0.5% v/v + zinc oxide nanoparticles (ZnO‐NPs) 1% v/v; (iv) Gly 1% v/v + T80 0.5% v/v + Eucalyptus radiata essential oil (EEO) 1% v/v; and (v) CS 1% w/v + Gly 1% v/v + T80 0.5% v/v + ZnO‐NPs 1% v/v + EEO 1% v/v.i

The stock solutions were prepared at 1 g 100 mL^−1^ for chitosan (1% w/v), 1 mL 100 mL^−1^ (1% v/v) for Gly, ZnO‐NPs, and EEO, and 0.5 mL 100 mL^−1^ (0.5% v/v) for T80. Serial two‐fold dilutions (1:2) were performed in triplicate across 12 steps. Thus, the first well contained 50% of the initial solution (e.g., 0.5% for CS), while the last well corresponded to 0.0244% of the 1% stock solution, equivalent to approximately 0.000244% v/v or 2.44 µL L^−1^ for CS, Gly, ZnO‐NPs, and EEO, and 1.22 µL L^−1^ for T80. These dilutions were used to determine the MIC and the MBC.

Wells containing only MHB and bacterial suspension, without the addition of experimental samples, served as positive growth controls. A standardised inoculum (0.5 McFarland, *ca* 1.5 × 10^8^ CFU mL^−1^) was diluted to 1.5 × 10^6^ CFU mL^−1^ in MHB and dispensed into both test and control wells. Plates were incubated at 37 °C for 24 h.

For MBC determination, aliquots from each well were streaked onto Mueller–Hinton agar and reincubated at 37 °C for 24 h. The MBC was defined as the lowest concentration at which no visible colonies were observed. MIC was determined by adding 30 μL of 1% 2,3,5‐triphenyltetrazolium chloride to each well, with colour change visually assessed as an indicator of metabolic activity.

#### *In vitro* antifungal effect against *L. theobromae*

The fungus *L. theobromae* was isolated from mangoes exhibiting symptoms of stem‐end rot, cultivated in a commercial orchard in Petrolina, Pernambuco, Brazil.

To evaluate the antifungal effect of the complete active coating formulation, 200 μL of the standardised solution was evenly distributed over the surface of Petri dishes (9 cm in diameter) containing potato dextrose agar (PDA) medium using a Drigalski spatula. For the control group, 200 μL of distilled water was applied under the same conditions. The plates were left open under a laminar‐flow hood for 15 min to allow surface drying. Subsequently, a 5 mm diameter mycelial disc, excised from the active margin of an *L. theobromae* colony, was placed at the centre of each Petri dish.

The plates were incubated at 25 °C under a 12 h photoperiod. After 3 days, colony diameters were measured along two perpendicular axes, and the average diameter was calculated. The experiment was conducted in a completely randomised design, with three replicate plates per group (control and treatment; *n* = 3); each plate was an experimental unit. Data were subjected to analysis of variance (ANOVA).

The percentage of inhibition of mycelial growth (PIMG) was calculated using the following equation:

$$\text{PIMG}=\frac{C-T}{C}\;\times \;100$$

where *C* is the average growth in the control group (distilled water) and *T* is the average growth in the treatment group (coating solution).

### Preparation and treatment of ‘Tommy Atkins’ mangoes

‘Tommy Atkins’ mangoes were harvested at commercial maturity in a commercial orchard located in Petrolina, Pernambuco, Brazil. A total of 200 fruits were selected based on uniform size, external appearance, ripening stage and absence of mechanical damage. After selection, the fruits were randomised to compose the experimental groups.

For surface sanitisation, the fruits were immersed for 5 min in a sodium hypochlorite solution prepared by diluting 5 mL of sodium hypochlorite solution (12% v/v) with distilled water to a final volume of 1 L. The sanitisation procedure was carried out using four 20 L plastic containers, each filled with the disinfectant solution as prepared above, totalling 100 mL of concentrated solution per container. After disinfection, the fruits were manually dried using paper towels.

Only the fruit assigned to the treated group (*n* = 60) received the active coating formulation, applied by manual spreading in two consecutive layers. Each layer was allowed to dry at room temperature (*ca* 25 °C, ambient airflow) until dry‐to‐the‐touch (no visible gloss transfer on gentle contact) before applying the next layer. Control mangoes were handled identically and received distilled water in place of the active coating, and additional uncoated fruits were used for baseline analysis (day 0).

**Figure 1:**
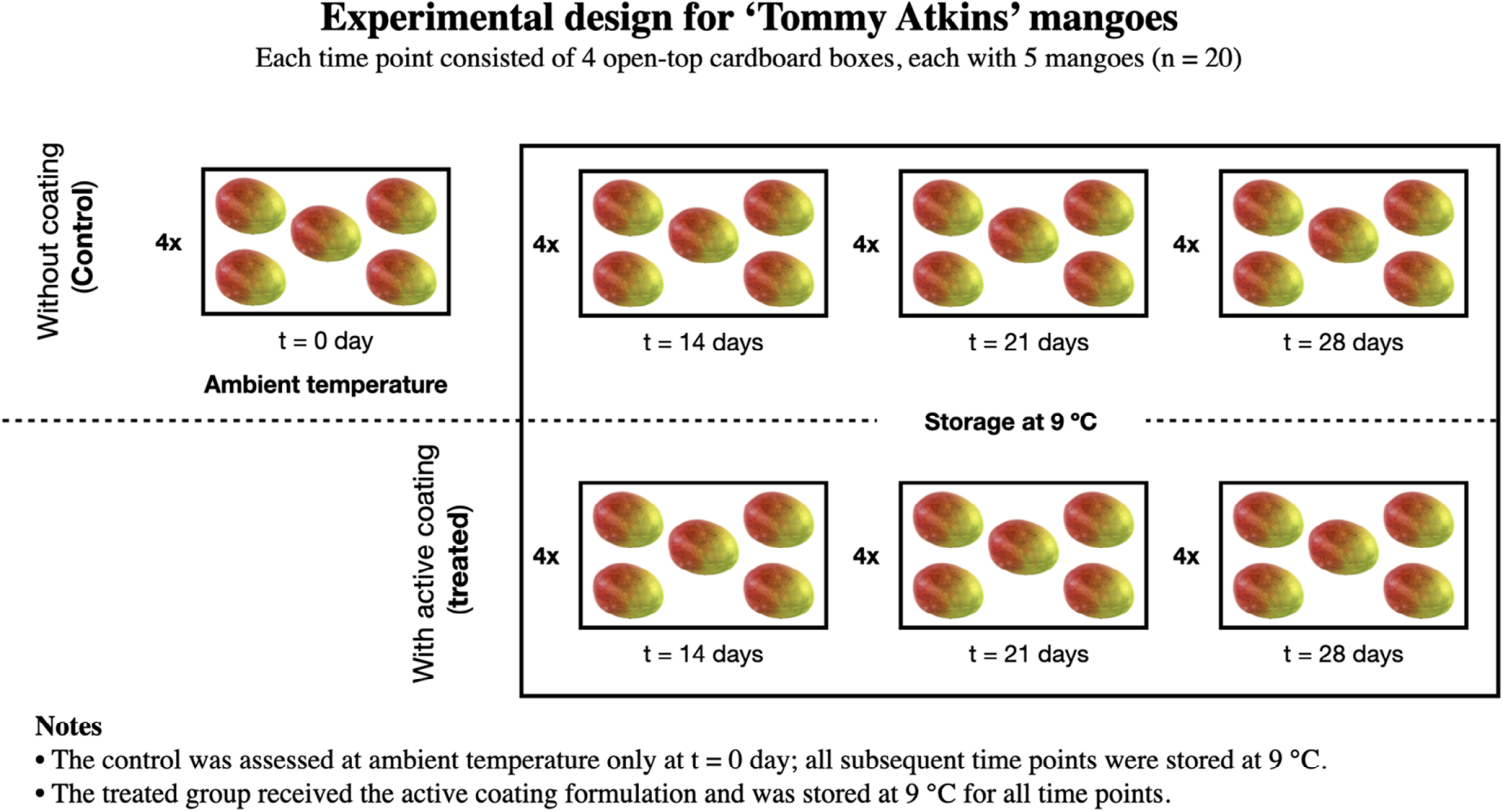
Experimental design and sampling scheme for ‘Tommy Atkins’ mangoes under control (without coating) and treated (with active coating) conditions.

In total, 140 mangoes were used in the experiment: 60 in the treated group, 60 in the control untreated group and 20 fruits that were analysed at harvest. The fruits were placed into 28 cardboard boxes, each containing five mangoes. Twelve boxes were allocated to the treated group, four for each storage period (14, 21 and 28 days), and another 12 to the control untreated group with the same distribution. Four additional boxes containing uncoated fruit were analysed at harvest. Fruits designated for storage were kept at 9 °C and were analysed at the end of each storage period at 14, 21 and 28 days. The remaining 60 fruits were not used experimentally. A schematic of the experimental workflow is provided (Fig. 1).

### Postharvest quality assessment: parameters and analytical methods

The postharvest quality of ‘Tommy Atkins’ mangoes was evaluated based on physical, chemical and physiological parameters that indicate fruit ripening, senescence and deterioration during storage. Analyses were performed at regular intervals, according to the previously described experimental design, using standardised analytical methods. The fruit were evaluated for weight loss, dry matter content, pulp firmness, skin and pulp colour, delta absorbance (DA; a non‐destructive ripening index derived from skin and pulp absorbance, where lower DA denotes more advanced ripening), total soluble solids (°Brix), titratable acidity, ascorbic acid (vitamin C), *β*‐carotene content and respiration rate. Detailed procedures are presented in the following subsections.

The fruit were analysed at harvest and after 14, 21 and 28 days of storage at 9 °C. The data were subjected to ANOVA and the means were compared using Tukey's test (5%).

#### Fresh weight loss

Before storage, fruit were weighed for each treatment group using a digital balance. The fruit were then stored and reweighed at the end of each storage period to determine mass loss over time. The percentage of fresh weight loss was calculated as:

$$\text{Weight loss}\;\left(\%\right)=\frac{\text{Initial weight}\left(g\right)-\text{Final weight}\left(g\right)}{\text{Initial weight}\left(g\right)}\;\times \;100$$

#### Physicochemical and visual properties

The firmness, skin and pulp colour and DA index of mangoes were evaluated as indicators of fruit ripening and quality. Pulp firmness was measured using a fruit hardness tester (model PTR‐300, Instrutherm, Brazil) equipped with a 6 mm diameter cylindrical probe. Measurements were taken by penetrating the probe 10 mm into the equatorial region of the fruit after skin removal, and results were expressed in newtons (N), representing the force required for penetration.

The skin and pulp colours were assessed using a digital colourimeter (Delta Vista 450G, Brazil). Colour values were recorded in the CIELAB colour space, expressed as L* (lightness, 0 = black to 100 = white), a* (green to red) and b* (blue to yellow). Skin and pulp colour measurements were taken at the equatorial region, on two opposite sides of each fruit. Skin colour was measured on the fruit surface, while pulp colour was measured on the inner pulp, near the seed, after cutting each side of the fruit.

The DA index, which estimates chlorophyll degradation and maturity level, was measured using a DA‐Meter (Turoni, Italy). The device evaluates the difference in absorbance between two specific wavelengths (670 and 720 nm), providing a non‐destructive index of fruit ripening. Measurements were taken at two equidistant points along the equatorial region of each fruit.

#### Physicochemical quality parameters

The physicochemical parameters evaluated in mango pulp included dry matter content, total soluble solids (°Brix), titratable acidity, ascorbic acid content and *β*‐carotene concentration.

Dry matter content was determined using composite pulp samples from five fruit per group, placed into pre‐labelled plastic cups. The cups were weighed empty and then after addition of the fresh sample. Later, the cups containing fresh fruit samples were placed in a forced‐air oven at 60 °C for 7 days until complete dehydration. After drying, the cups were weighed again. The percentage of dry matter was calculated as the ratio between the mass of the dehydrated sample and the mass of the fresh sample, multiplied by 100.

Total soluble solids were measured from crude pulp juice using a digital refractometer (Hybrid PAL‐BX ACID F5, Atago, Brazil), with results expressed in °Brix. A single representative reading was recorded per group.

Titratable acidity was determined by titrating 1 g of pulp juice diluted in 50 mL of distilled water with 0.1 N sodium hydroxide to pH 8.1, using an automatic titrator (Titrino Plus 848, Metrohm, Brazil). Results were expressed as percentage of citric acid.

Ascorbic acid content was quantified by titration with Tillman's reagent (2,6‐dichlorophenolindophenol, 0.02%). For this analysis, 5 mL of juice was diluted in 100 mL of 0.5% oxalic acid. Then, 1 mL of this solution was further diluted in 49 mL of distilled water and titrated until the appearance of a persistent light pink colour for 15 s. Results were expressed as milligrams of ascorbic acid per 100 g of juice.

*β*‐Carotene concentration was determined according to Nagata and Yamashita,^14^ using a hexane–acetone extraction solution (60:40, v/v). A 10 mg aliquot of juice was mixed with 20 mL of the solvent solution in a Falcon tube, homogenised in a vortex mixer for 1 min and left to rest for another 1 min. The supernatant was transferred to test tubes, and absorbance was measured in glass cuvettes at 663, 645, 505 and 453 nm. *β*‐Carotene concentration was calculated as:

$$\beta -\text{Carotene}\left(\mathrm{mg}\;{\left(100\;\mathrm{mL}\right)}^{-1}\right)=\begin{array}{l} \left(0.216\;\times \;A_{663}\right)-\left(1.22\;\times \;A_{645}\right)\\ -\;\left(0.304\;\times \;A_{505}\right)+\left(0.452\;\times \;A_{453}\right) \end{array}$$

The values were multiplied by 1000 to be expressed in μg (100 mL)^−1^.

#### Respiration rate

Mango respiration rate was evaluated in the control and treated groups at harvest and after 14, 21 and 28 days of storage (replication scheme as outlined in Section Postharvest quality assessment: parameters and analytical methods). For each group, the total mass of five fruit per replication was recorded using a semi‐analytical balance. The fruit were then placed in properly labelled plastic containers, which were sealed and maintained at 20 °C for 2 h to allow CO_2_ accumulation inside the headspace; this assay temperature was selected to provide a robust CO_2_ signal within the accumulation period and comparability with ambient‐temperature respiration assays, and does not represent the storage temperature (9 °C). After this period, the CO_2_ concentration was determined with a portable gas analyser (Felix F‐950, Felix Instruments, USA). These data were used to calculate the respiration rate, expressed as mol CO_2_ kg^−1^ h^−1^.

### Inoculation protocol and evaluation of anthracnose development in mangoes

‘Tommy Atkins’ mangoes were purchased from a local market in Petrolina, PE, Brazil. The fruit were washed under running water to remove surface debris, followed by surface sterilisation with 70% ethanol. After sterilisation, the fruit were distributed into three trays, each containing eight fruits (total 24), allowing three replicates per treatment across eight experimental groups (Table 1).

Eight treatment groups were used (Table 1): T1, 1% CS (in 1% acetic acid); T2, composite formulation (1% CS + 1% glycerol + 0.25% Tween 80 + 0.5% ZnO‐NPs + 0.5% EEO, in 1% acetic acid); T3, 0.25% Tween 80 (in water); T4, 1% acetic acid; T5, 0.5% ZnO‐NPs (in water); T6, 0.25% Tween 80 + 0.5% EEO (in water); T7, positive control (sterile distilled water); T8, negative control (sterile distilled water).

Each mango was mechanically wounded in four predefined areas using a custom‐made device equipped with seven parallel needles (1 mm in length), ensuring consistent lesion depth across all samples. A 200 μL aliquot of the corresponding treatment solution was applied to each wound and gently spread using gloved fingers to maintain aseptic conditions. The fruit were then left uncovered at approximately 25 °C (±3 °C) to dry.

The fungal inoculum was prepared by adding sterile distilled water to Petri dishes containing *Colletotrichum* sp. colonies grown on PDA, followed by gentle scraping to facilitate conidial release. The suspension was filtered through sterile gauze, quantified using a Neubauer haemocytometer under light microscopy and adjusted to 1 × 10^5^ spores mL^−1^. Two hours after treatment application, 50 μL of the spore suspension was inoculated onto each wounded site for T1–T7; T8 (negative control) was not inoculated and was otherwise handled identically (wounding and application of sterile distilled water).

The fruit were placed in plastic containers sealed with PVC film to maintain high relative humidity and were incubated at approximately 25 °C (±3 °C) for 6 days; this environment was selected to favour anthracnose development and reflects ambient bench‐top conditions (relative humidity was not instrumentally recorded). Lesion development was monitored daily, and photographic records were taken to evaluate anthracnose progression and treatment efficacy.

To quantify disease severity, necrotic lesions were measured using ImageJ software (National Institutes of Health, Bethesda, MD, USA). The lesion area was calculated from the average of the four inoculated sites per fruit and used to generate the disease progress curve. From these data, the area under the disease progress curve (AUDPC) was calculated according to Shaner and Finney,^15^ allowing statistical comparison of treatment efficacy across the incubation period.

### Severity and aetiology of postharvest decay

After treatment application, the fruit were stored at 9 °C for 28 days to simulate commercial shipping. The severity of postharvest decay was assessed visually, as the percentage of symptomatic surface per fruit based on whole‐fruit inspection.

At the end of storage, symptomatic fruit were selected for mycological isolation. Small tissue fragments were aseptically excised from lesion margins and transferred to Petri dishes containing PDA. Plates were incubated at 25 °C and monitored daily for fungal growth. The predominant pathogen was identified from colony characteristics on PDA and by microscopic observation of reproductive structures, particularly conidia (shape and septation) under light microscopy; no molecular confirmation was performed.

The experiment followed a randomised block design with four replications, each experimental unit consisting of one fruit. Based on the visual severity estimates, disease progress curves were constructed and the AUDPC was calculated following Shaner and Finney.^15^ AUDPC data were analysed by ANOVA, and treatment means were compared using the *F*‐test at the 5% significance level (*P* ≤ 0.05) using Sisvar software.^16^

## RESULTS AND DISCUSSION

### *In vitro* antimicrobial and antifungal activity of bioactive formulation

The antimicrobial potential of the coating components was evaluated against *S. aureus* (ATCC 25923) and *E. coli* (ATCC 25922), as presented in Table 2. Most treatments exhibited lower MIC and MBC values against *S. aureus*, indicating greater susceptibility of the Gram‐positive strain. The formulation containing glycerol, Tween 80 and EEO, in the absence of CS and ZnO‐NPs, showed the highest MIC and MBC values (25%) for both strains, reflecting low antimicrobial efficacy without the key bioactive agents.

**Table 2:** Determination of MIC and MBC of the polymer and/or additives present in the coating to identify the active components responsible for the antimicrobial activity against *Staphylococcus aureus* (ATCC 25923) and *Escherichia coli* (ATCC 25922)

Sample^a^	ATCC 25923		ATCC 25922	
	MIC (%)	MBC (%)	MIC (%)	MBC (%)
[CS 1.0%]	6.25	6.25	12.50	12.50
[CS 1.0%/Gly 1.0%]	6.25	6.25	12.50	12.50
[Gly 1.0%/T80 0.5%/ZnO‐NPs 1.0%]	6.25	6.25	12.50	12.50
[Gly 1.0%/T80 0.5%/EEO 1.0%]	25.00	25.00	25.00	25.00
[CS 1.0%/Gly 1.0%/T80 0.5%/ZnO‐NPs 1.0%/EEO 1.0%]	6.25	6.25	6.25	6.25

^a^ All sample preparations were made in 1.0% acetic acid.

CS, chitosan; EEO, *Eucalyptus radiata* essential oil; Gly, glycerol; T80, tween 80; ZnO‐NPs, zinc oxide nanoparticles.

Key endpoints (% v/v) were: for *S. aureus*, MIC = MBC 6.25 in all formulations containing CS or ZnO‐NPs, but 25.00 for the Gly/T80/EEO mixture; for *E. coli*, only the complete formulation reached 6.25/6.25, whereas CS, CS + Gly and Gly/T80/ZnO‐NPs were 12.50/12.50, and Gly/T80/EEO was 25.00/25.00 (Table 2).

Conversely, the complete formulation, consisting of 1.0% CS, 1.0% glycerol, 0.5% Tween 80, 1.0% ZnO‐NPs and 1.0% EEO, was the only treatment to achieve equally low MIC and MBC values (6.25%) for both bacteria, indicating broad‐spectrum efficacy and strong synergistic action against these pathogens. While formulations containing CS or ZnO‐NPs (without CS) were effective against *S. aureus*, only the complete formulation significantly inhibited *E. coli*, reinforcing the importance of multicomponent synergy against Gram‐negative bacteria.

These findings are supported by previous studies reporting enhanced antimicrobial effects when essential oils are combined with biopolymers or nanomaterials, particularly against *E. coli*.^17^, ^18^ The outer membrane of *E. coli*, rich in lipopolysaccharides, poses a barrier to many antimicrobials, requiring more potent or synergistic strategies for effective inhibition.

Although EEO alone showed limited activity, its contribution within the full formulation is likely due to synergistic interactions with CS and ZnO‐NPs. Key constituents of EEO, such as 1,8‐cineole, limonene, *α*‐terpineol and *α*‐pinene, are known to disrupt bacterial membranes, promote leakage of intracellular contents and generate oxidative stress.^19^, ^20^, ^21^ Moreover, previous studies have shown that the incorporation or encapsulation of essential oils improves their antimicrobial stability and efficacy.^22^

Glycerol, added as a plasticiser, did not interfere with antimicrobial activity and likely contributed to film integrity. ZnO‐NPs, in turn, played a critical role in both bacteriostatic and bactericidal activity, especially against *S. aureus*, consistent with reports of higher sensitivity of Gram‐positive bacteria to ZnO‐NPs.^23^, ^24^ Their antimicrobial action involves the release of Zn^2+^ ions, membrane interaction and the generation of ROS, resulting in cellular damage and death; this behaviour is consistent with reports for ZnO‐NPs in fruit‐coating systems, where growth inhibition has been attributed to ROS generation at the cell surface in addition to Zn^2+^ release.^9^

In addition to its antibacterial efficacy, the formulation was also evaluated for antifungal activity against *L. theobromae*, a major postharvest pathogen of tropical fruits. After determining low MIC/MBC endpoints *in vitro*, the composition was adjusted to reduce the load of bioactive additives whilst maintaining activity: the concentrations of ZnO‐NPs and EEO were halved to 0.5% each, Tween 80 was kept proportional at 0.25% and CS and glycerol were maintained at 1.0%. This adjusted formulation (CS 1.0%, Gly 1.0%, T80 0.25%, ZnO‐NPs 0.5%, EEO 0.5%) was then used in the subsequent antifungal assays.

**Figure 2:**
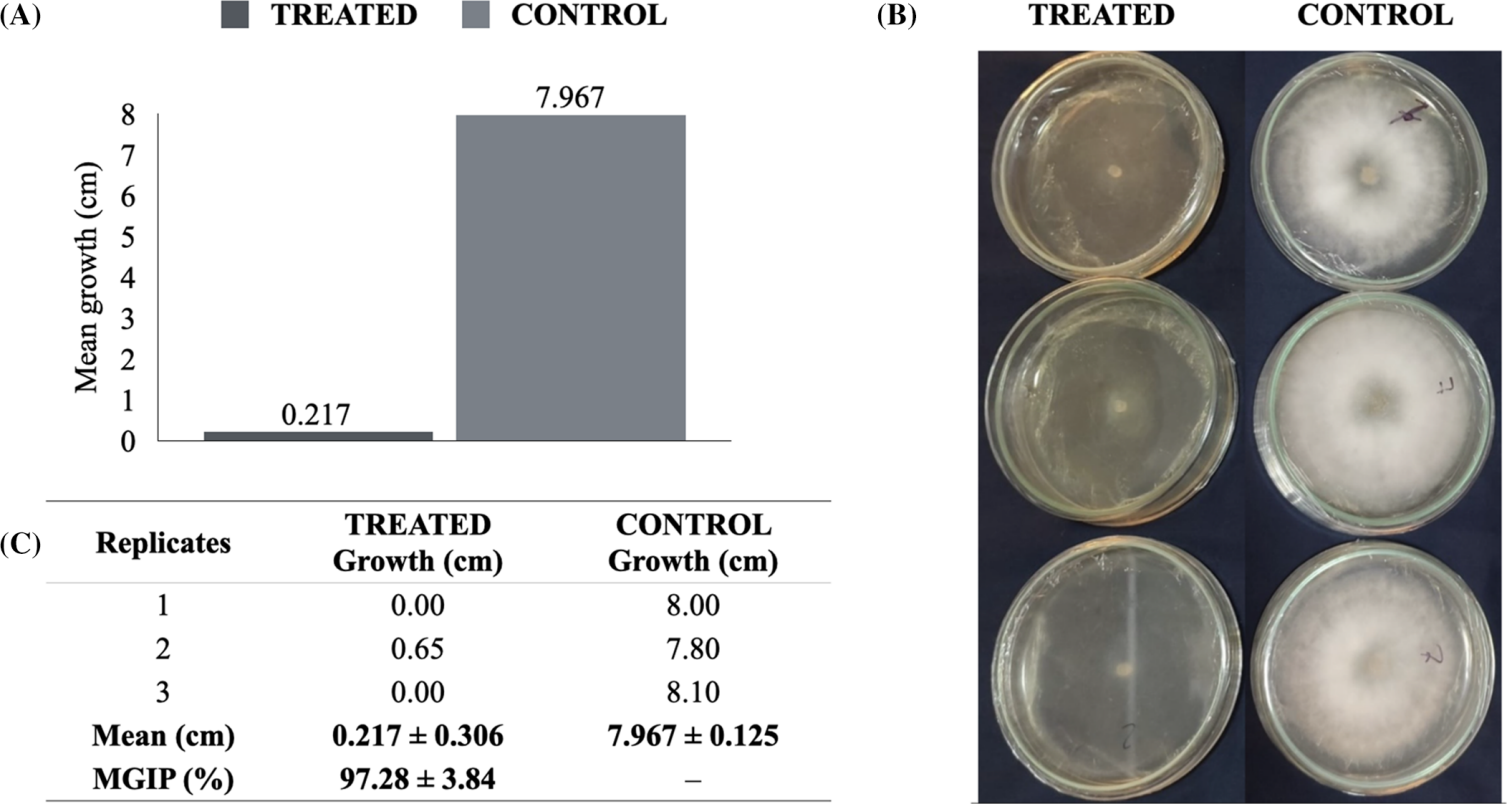
*In vitro* inhibition of *Lasiodiplodia theobromae* by the bioactive coating formulation: (A) mean mycelial growth in treated and control groups; (B) visual representation of fungal development on Petri dishes after 3 days of incubation at 25 °C; (C) mean growth values and PIMG.

Statistical analysis revealed a significant difference in mycelial growth between treated and control groups (ANOVA: *F* = 1026.21; *P* < 0.001). As shown in Fig. 2, the mean mycelial growth in the control group was 7.967 ± 0.125 cm, while the treated group exhibited only 0.217 ± 0.306 cm, which corresponds to just 2.72% of the growth observed in the untreated group. This reflects a PIMG of 97.28 ± 3.84% (Fig. 2(C)), confirming the strong antifungal activity of the tested coating against *L. theobromae*. These ANOVA results corroborate the superior antifungal performance of the adjusted formulation relative to the control and align with the large reduction in mean colony diameter and the high inhibition percentage.

CS served as the polymeric matrix in the formulation and is well documented for its antifungal properties. Previous studies have reported inhibition rates ranging from 70% to 90% against *L. theobromae*, *Alternaria alternata* and *Colletotrichum gloeosporioides*.^25^, ^26^, ^27^ Its antifungal mechanism is primarily attributed to the interaction of positively charged amino groups with negatively charged fungal cell membranes, resulting in ionic imbalance (K^+^ and Ca^2+^), altered permeability, hyphal deformation and eventual cell collapse.^28^ Additionally, CS can form a physical barrier on fruit surfaces, preventing fungal penetration.

The high pathogen inhibition rate observed in this study (97.28%) suggests a synergistic antifungal effect resulting from the combination of CS with EEO and ZnO‐NPs. This hypothesis is supported by previous studies demonstrating enhanced efficacy of similar pathogen‐control approaches. For instance, CS/ZnO nanocomposites inhibited *Aspergillus* sp. growth by 93%,^29^ and CS/ZnO/essential‐oil coatings showed 85.91% *in vitro* inhibition of *Penicillium italicum* and 79.86% *in vivo* on tangerines.^8^ Likewise, CS‐based sachets containing microencapsulated EEO reduced *P. chrysogenum* growth by 96%, significantly decreasing lesion size in inoculated peaches.^30^ The antifungal mechanism of ZnO‐NPs involves the generation of ROS and the release of Zn^2+^ ions, which together induce oxidative stress and compromise membrane integrity, culminating in cell death.^31^ In parallel, the lipophilic nature of essential oils enables their integration into fungal membranes, disrupting respiration, membrane proteins and ion transport, ultimately increasing permeability and leading to cellular collapse.^32^, ^33^ We note that synergy was inferred qualitatively; quantitative interaction indices (e.g. FICI from checkerboard assays) were not determined here and will be addressed in future work.

In summary, the complete formulation (CS, glycerol, Tween 80, ZnO‐NPs and EEO) achieved the lowest MIC/MBC values for both *S. aureus* and *E. coli* and produced a marked reduction in mycelial growth of *L. theobromae in vitro*. Chitosan or ZnO‐NPs alone were mainly active against the Gram‐positive strain, whereas inhibition of *E. coli* required the full multicomponent system, indicating a synergistic effect. These findings indicate potential applicability of this edible coating to postharvest disease management in mangoes, complementing cold‐chain practices and potentially reducing reliance on synthetic fungicides.

### Impact of coating on postharvest quality preservation of ‘Tommy Atkins’ mangoes

The application of the bioactive coating in the defined formulation of 1.0% CS, 1.0% glycerol, 0.25% Tween 80, 0.5% ZnO‐NPs and 0.5% EEO significantly influenced various quality attributes of ‘Tommy Atkins’ mangoes during storage at 9 °C. Although no statistically significant differences were observed in weight loss (Table 3), a trend towards lower cumulative weight loss was noted in coated fruit at the end of 28 days of storage (3.75%) compared with uncoated fruit (4.96%). This result suggests that the coating did not increase water loss and may have contributed to reduced transpiration, although the high variability in the data (CV = 27.74%) limited the detection of statistical differences.

**Table 3:** Weight loss, delta absorbance, pulp firmness and skin and pulp colour parameters (*L*, *a*, *b*) of uncoated and coated ‘Tommy Atkins’ mangoes at harvest and during storage at 9 °C for up to 28 days*

Coating	Weight loss (%)	Delta absorbance	Firmness (N)	Skin colour			Pulp colour		
				L	a	b	L	a	b
	Day 0 at 20 °C (at harvest)								
Mean	0	2.08	12.14	50.77	−5.33	33.11	69.22	14.52	63.27
SD	0	0.017	1.57	1.12	0.60	2.25	1.88	1.13	3.32
	14 days of storage at 9 °C								
Uncoated	1.60^A^ ^a^	1.87^A^ ^a^	8.88^A^	49.6^A^	−3.12^A^	36.2^A^	71.3^A^	15.1^A^	60.7^A^
Coated	1.72^A^	2.03^A^	9.35^A^	49.6^A^	−5.33^B^	34.5^A^	70.7^A^	14.1^A^	60.5^A^
CV (%)	7.09	7.59	29.9	5.19	−16.6	4.75	1.66	10.4	4.01
	21 days of storage at 9 °C								
Uncoated	2.77^A^	1.90^A^	6.79^A^	52.9^A^	−1.32^A^	36.9^A^	66.3^B^	14.1^A^	60.9^A^
Coated	2.73^A^	2.01^A^	10.52^A^	48.6^B^	−3.62^A^	36.9^A^	69.1^A^	14.7^A^	63.0^A^
CV (%)	9.02	3.27	30.7	3.61	−54.8	5.01	1.80	8.45	3.07
	28 days of storage at 9 °C								
Uncoated	4.96^A^	1.77^B^	3.42^B^	50.3^A^	−0.40^A^	38.8^A^	64.9^A^	12.9^A^	54.4^B^
Coated	3.75^A^	1.91^A^	7.30^A^	49.9^A^	−3.98^B^	35.4^B^	70.7^A^	14.1^A^	61.1^A^
CV (%)	27.74	2.82	24.0	2.58	−54.6	3.17	5.64	10.1	3.55

Sample size per treatment per time‐point: *n* = 20 fruits; assay‐specific subsampling and measurement details are provided in Sections Fresh weight loss and Physicochemical and visual properties.

^a^ Means followed by the same letter (A,B) are statistically equal according to Tukey's test (5%).

Pulp firmness was one of the most affected quality parameters. At the end of storage, coated fruit had higher firmness (7.30 N) than control fruit (3.42 N) (Table 3). This difference indicates that the bioactive coating helped delay the softening process, which is a desirable effect in climacteric fruit such as mangoes, where firmness loss is associated with ripening and the activity of cell‐wall‐degrading enzymes.^34^

Colour parameters were also affected by the treatment. The skin of coated fruit showed significantly lower a* (−3.98) and b* (35.4) values, indicating better preservation of green hues and reduced progression towards yellowing, respectively. In contrast, uncoated fruit exhibited higher a* (−0.40) and b* (38.8) values, reflecting the typical yellowing associated with ripening (Table 3). In the pulp, the b* value was also higher in coated fruit (61.1) compared with uncoated fruit (54.4), suggesting retention of the vibrant colour typical of fresh pulp. These findings align with the role of the coating in reducing fruit metabolic activity and ripening, resulting in lower rates of chlorophyll degradation and carotenoid synthesis, as described by Baldwin *et al*.^35^

DA analysis further supports the coating's ripening‐delay effect. After 28 days of cold storage, coated fruit showed a mean DA value of 1.91, significantly higher than that of the control group (1.77) (Table 3). For context, DA decreases as ripening advances; higher DA denotes greener/less ripe fruit with greater chlorophyll and lower carotenoid/lycopene contents, whereas lower DA reflects more advanced ripening.^36^ These results indicate that the tested coating effectively preserved the physiological integrity of the fruit by delaying both ripening and senescence‐related processes.

Regarding the physicochemical parameters evaluated (Table 4), total soluble solids (°Brix) were significantly lower in coated fruit (9.04%) compared with uncoated fruit (11.40%) after 14 days of cold storage. This result suggests a slower ripening process in coated fruit, since soluble sugar accumulation is generally associated with starch degradation during ripening.^37^ However, this difference was not significant in later evaluations, possibly due to the ongoing endogenous metabolism of the fruit over time.

**Table 4:** Dry matter, total soluble solids (°Brix), titratable acidity (% citric acid), ascorbic acid (mg 100 g^−1^), β‐carotene (μg 100 mL^−1^), and respiration rate (mol CO_2_ kg^−1^ h^−1^) of uncoated and coated ‘Tommy Atkins’ mangoes at harvest and during storage at 9°C for 28 days.

Coating	Dry matter (%)	Soluble solids (%)	Titratable acidity (%)	Ascorbic acid (mg 100 g^−1^)	β‐Carotene (μg 100 mL^−1^)	Respiration rate (mol CO_2_ kg^−1^ h^−1^)
	Day 0 at 20 °C (at harvest)					
Mean	11.09	9.32	0.712	15.55	0.128	1.438
SD	0.89	0.53	0.147	4.61	0.183	0.094
	14 days of storage at 9 °C					
Uncoated	10.8^a^ ^*^	11.40^a^	0.49^a^	12.1^a^	0.069^a^	0.405^a^
Coated	10.7^a^	9.04^b^	0.71^a^	12.8^a^	0.084^a^	0.288 ^b^
CV (%)	5.61	2.22	25.3	17.1	41.5	7.46
	21 days of storage at 9 °C					
Uncoated	11.4^a^	11.4^a^	0.41^a^	12.1^a^	0.040^a^	0.365^a^
Coated	10.4^b^	10.8^a^	0.64^a^	12.8^a^	0.087^a^	0.416^a^
CV (%)	4.96	5.74	30.3	17.1	75.2	10.03
	28 days of storage at 9 °C					
Uncoated	9.6^a^	10.6^a^	0.31^a^	12.1^a^	0.037^a^	0.483^a^
Coated	9.7^a^	10.8^a^	0.54^a^	13.5^a^	0.044^a^	0.504^a^
CV (%)	5.88	6.79	52.3	22.7	24.5	12.33

*Note*: Sample size per treatment per time‐point: *n* = 20 fruits; assay‐specific subsampling and measurement details are provided in Sections 2.5.3–2.5.4.

^*^ Means followed by the same letter are statistically equal according to Tukey's test (5%).

Respiration rate also highlighted the coating's effect on inhibiting fruit metabolism during cold storage. Uncoated fruit exhibited an early respiratory peak of 0.405 mol CO_2_ kg^−1^ h^−1^ at 14 days, whereas coated fruit reached a similar value (0.416 mol CO_2_ kg^−1^ h^−1^) only at 21 days (Table 4). The seven‐day delay in the respiration peak suggests that the coating effectively inhibited fruit metabolic activity associated with the respiration rise characteristic of climacteric fruit. According to Parente *et al*.,^38^ coatings that act as semipermeable barriers can modify the atmosphere inside the fruit, reducing oxygen and increasing carbon dioxide levels, as well as limiting ethylene production, thereby delaying ripening and senescence‐related processes. This behaviour is supported by Parente *et al*.,^11^, ^39^ who associate lower respiration with extended shelf life and reduced postharvest losses. For broader relevance, values can be converted to mg CO_2_ kg^−1^ h^−1^ by multiplying by 44 010 mg mol^−1^ (e.g. 0.405 mol CO_2_ kg^−1^ h^−1^ ≈ 17 824 mg CO_2_ kg^−1^ h^−1^).

On the other hand, no statistically significant differences were observed in dry matter, titratable acidity, ascorbic acid or *β*‐carotene content between groups throughout the storage period (Table 4). This stability may reflect the moderating effect of cold storage (9 °C) on metabolic turnover, limiting treatment discrimination for these compositional attributes under the present conditions.^40^

Thus, the results demonstrate that the tested formulation had a positive effect on the postharvest quality preservation of ‘Tommy Atkins’ mangoes. The observed benefits, including firmness retention, delayed skin and pulp colour changes, a higher DA index and a postponed respiratory peak, reinforce the potential of the coating as an effective fruit‐preservation strategy. The combination of CS, EEO and ZnO‐NPs may have acted both as a physical barrier and as a bioactive system, modulating physiological and biochemical processes, as also reported by Elsabee and Abdou.^41^ These outcomes are consistent with reports that edible coatings, including CS‐based and polysaccharide–wax systems, can slow respiration and help maintain firmness during cold storage, thereby preserving quality.^40^, ^41^

### Bioactive treatments for anthracnose control in ‘Tommy Atkins’ mangoes

**Figure 3:**
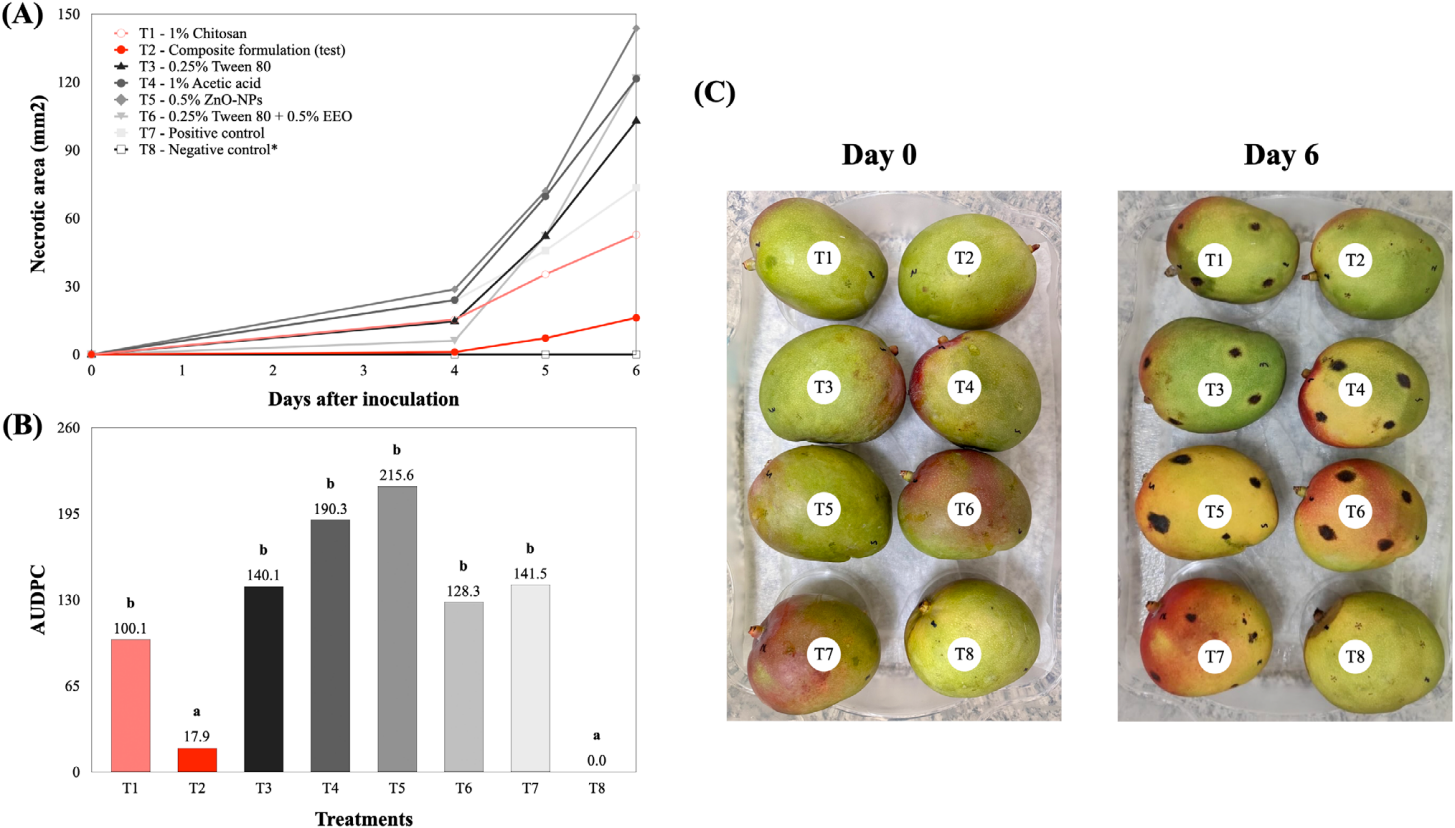
Effect of treatments on anthracnose development in ‘Tommy Atkins’ mangoes inoculated with *Colletotrichum* sp. (A) Disease progress curves based on the average necrotic lesion area per fruit, calculated from four lesions per fruit across three replications. (B) AUDPC for each treatment; T2 (composite formulation) exhibited the lowest AUDPC (17.9). Bars with the same letter do not differ significantly according to the Scott–Knott test (*P* ≤ 0.05). (C) Visual appearance of mangoes at day 0 (pre‐inoculation) and day 6 after incubation at 20 °C, showing disease symptom progression in fruit subjected to each treatment. Treatment T8 corresponds to the negative control (non‐inoculated), indicated by an asterisk (*) in (A).

The effectiveness of the tested treatments in controlling the progression of anthracnose symptoms in ‘Tommy Atkins’ mangoes inoculated with *Colletotrichum* sp. is presented in Fig. 3, based on both quantitative and visual evaluations. The disease progress curves (Fig. 3(A)) revealed distinct patterns of necrotic lesion development among treatments over 6 days of incubation at 25 °C (± 3 °C). Among all treatments, only the defined bioactive formulation (T2), consisting of 1.0% CS (in 1% acetic acid), 1.0% glycerol, 0.25% Tween 80, 0.5% ZnO‐NPs and 0.5% EEO, significantly reduced lesion expansion throughout the incubation period.

This result is corroborated by the values of the AUDPC (Fig. 3(B)), where T2 exhibited the lowest value (17.9), differing significantly from all other treatments (T1 and T3 to T6), which were statistically similar to the negative control (T8; not inoculated). Treatments T1 (1% CS in 1% acetic acid) and T6 (0.25% Tween 80 + 0.5% EEO) also promoted a slight reduction in disease progression, with AUDPC values of 100.1 and 128.3, respectively, compared with the positive control T7 (141.5). Treatments based on individual components, such as Tween 80 (T3, 140.1), acetic acid (T4, 190.3) and ZnO‐NPs (T5, 215.6), showed greater disease severity, indicating that isolated applications were less effective than the combined formulation.

These quantitative findings are supported by the visual assessment (Fig. 3(C)), where fruit treated with the composite formulation remained visually healthy, similar to the negative control (T8). In contrast, fruit subjected to the other treatments, particularly T1 and T3 to T7, exhibited typical anthracnose symptoms, such as dark, circular lesions of varying sizes, confirming the limited efficacy of these formulations. Treatment T8, which was not inoculated with *Colletotrichum* sp., showed no signs of disease, validating the experimental design and confirming the pathogenicity of the inoculum.

The superior performance of the composite formulation suggests a synergistic interaction among its bioactive components, particularly between CS, ZnO‐NPs and EEO, forming an effective physical and biochemical barrier against fungal infection. This synergy likely results from the combination of multiple antimicrobial mechanisms, including disruption of the fungal cell membrane, generation of ROS and inhibition of spore germination. Similar effects were reported by Gowda and Sriram,^42^ who highlighted the enhanced antifungal activity of CS combined with metallic nanoparticles, and by Burt,^43^ who described the broad‐spectrum antimicrobial action of essential oils and ZnO‐NPs against phytopathogens.

Furthermore, the superior efficacy of the composite formulation compared with the CS treatment alone is consistent with previous studies indicating that multifunctional formulations may not only directly inhibit pathogen development but also induce defence responses in plant tissues.^44^, ^45^ Therefore, the tested formulation represents a promising natural alternative for extending postharvest shelf life and reducing fungal decay in mangoes, particularly under storage conditions, thereby minimising the need for synthetic fungicides.

### Evaluation of disease progression and fungal identification in postharvest mango decay under coating treatment

**Figure 4:**
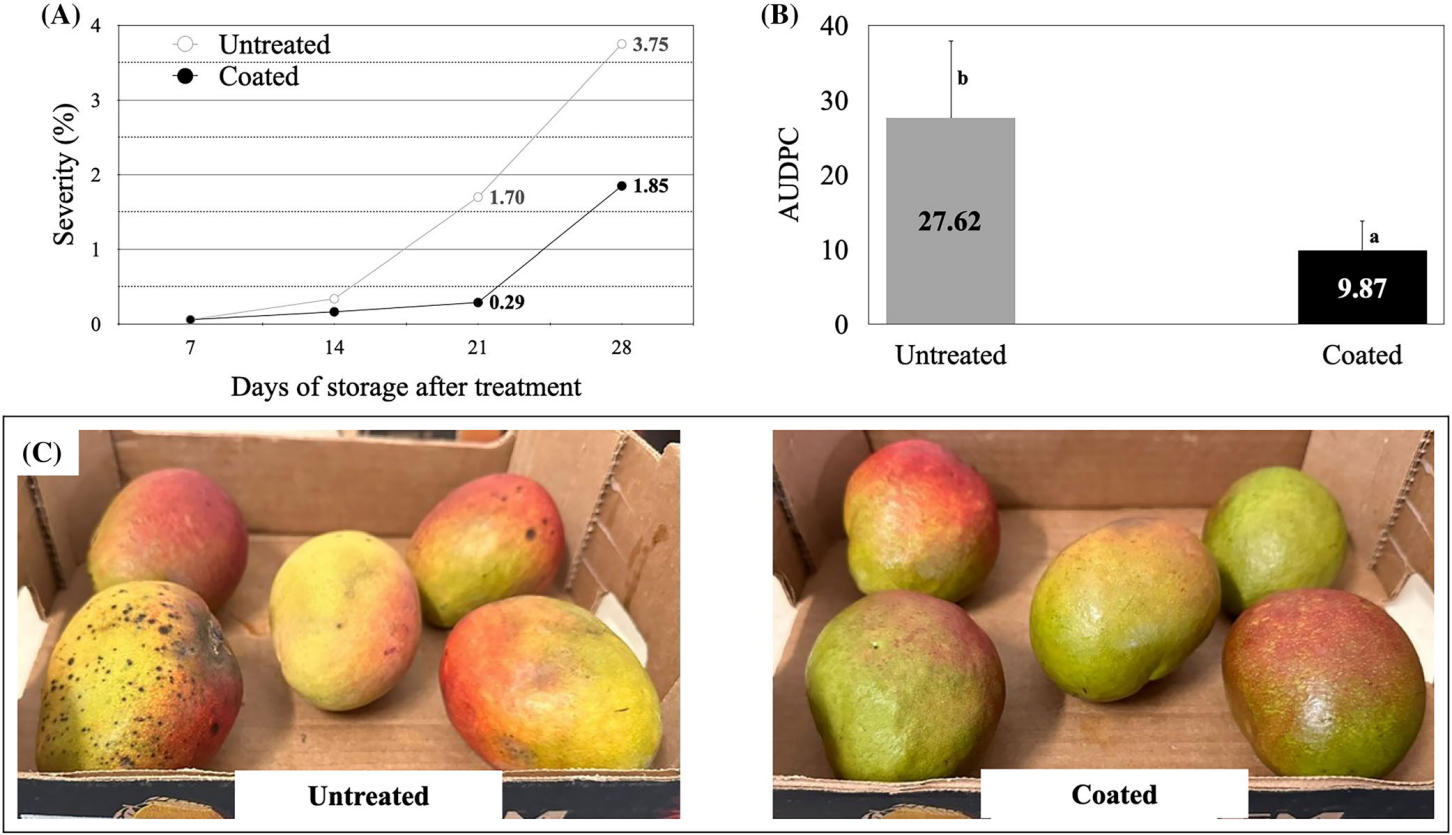
Effect of coating treatments on disease progression in ‘Tommy Atkins’ mangoes stored at 9 °C. (A) Disease severity progress curves. (B) AUDPC and anthracnose (*Colletotrichum* sp.) severity; mean AUDPC values: coated fruit = 9.87, uncoated fruit = 27.62. (C) Images of mangoes after 28 days of cold storage, with and without coating treatment. Bars with different letters indicate significant differences according to the *F*‐test (*P* ≤ 0.05).

The application of the defined bioactive coating formulation (1.0% CS, 1.0% glycerol, 0.25% Tween 80, 0.5% ZnO‐NPs and 0.5% EEO), as defined in Section *In vitro* antimicrobial and antifungal activity of the bioactive formulation, had a significant effect on mitigating postharvest decay symptoms in ‘Tommy Atkins’ mangoes during storage at 9 °C for 28 days. As shown in Fig. 4(A), the disease progression curve revealed a substantial increase in lesion severity in uncoated fruit, especially after day 14. By day 21, uncoated fruit reached a severity of 1.70, compared with just 0.29 in coated fruit. At the end of the storage period (day 28), the severity in uncoated fruit further increased to 3.75, while coated fruit reached only 1.85. These data indicate that the accumulation of necrotic symptoms in uncoated fruit was approximately twice as high as that observed in coated fruit, demonstrating the coating's ability to delay both the onset and progression of decay.

This trend was quantitatively confirmed by the AUDPC, as shown in Fig. 4(B). Coated fruit exhibited a mean AUDPC value of 9.87, while uncoated fruit reached 27.62. This represents a reduction of approximately 64.3% in disease progression due to the coating treatment. The statistical difference between treatments, indicated by distinct letters above the bars, was confirmed by the *F*‐test (*P* ≤ 0.05). These results suggest that the coating effectively reduced fungal development, likely through the combination of a physical barrier and bioactive components.

According to Romanazzi and Moumni,^46^ CS‐based coatings can act not only as semipermeable barriers, but also as natural antifungal agents, inhibiting the development of *Colletotrichum* sp., the pathogen commonly associated with mango anthracnose. In the present study, CS likely formed a protective film on the fruit surface, limiting oxygen diffusion and fungal colonisation. Its antifungal properties may have been enhanced by the synergistic effects of EEO and ZnO‐NPs, which contribute to antimicrobial action through the release of Zn^2+^ ions and the generation of ROS.^12^

Visually, the effectiveness of the coating was further supported by fruit appearance after 28 days. As shown in Fig. 4(C), coated fruit exhibited fewer lesions and better skin colour, with no visible signs of advanced decay, indicating preserved commercial quality and extended shelf life.

**Figure 5:**
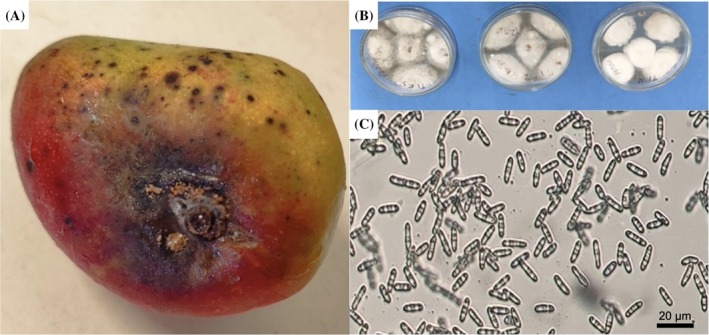
Isolation and identification of the predominant fungal pathogen in mangoes. (A) Visible symptoms on the fruit surface, including sporulation characteristic of *Colletotrichum* sp. (B) Isolation on PDA. (C) Light micrograph of *Colletotrichum* conidia under bright‐field microscopy (total magnification: ×400; scale bar: 20 μm, generated by microscope software).

In order to confirm the aetiological agent responsible for the symptoms, microbiological isolation was performed on symptomatic fruit from both treatments. As shown in Fig. 5(A), typical stem‐end rot symptoms, such as localised darkening and evident sporulation, were observed in infected fruit. Tissue samples from the lesions were cultured on PDA, leading to the development of colonies with morphological characteristics consistent with fungi of the genus *Colletotrichum* (Fig. 5(B)). Microscopic analysis revealed fusiform and septate conidia (Fig. 5(C)), characteristic of this genus, corroborating previous studies that identified *Colletotrichum* as the principal pathogen involved in postharvest decay of mangoes in northeastern Brazil.^47^

Therefore, these data indicate that the tested bioactive coating may represent a promising strategy for postharvest decay control, combining physical barrier effects with biochemical mechanisms conferred by its components. CS, due to its recognised antifungal properties and film‐forming ability,^12^ in association with the antimicrobial potential of EEO and ZnO‐NPs,^31^ may have contributed to the suppression of pathogen growth and development. Although studies combining all three agents are relatively scarce, previous research highlights the synergistic effects between essential oils and nanoparticles or biopolymers as promising for active coating applications.^48^

## CONCLUSIONS

This study shows that a CS‐based coating enriched with ZnO‐NPs and EEO is a promising nature‐derived strategy for maintaining quality and reducing postharvest losses of ‘Tommy Atkins’ mangoes. Quantitatively, the composite formulation achieved 97.28% inhibition of *Lasiodiplodia theobromae in vitro*, delivered the lowest AUDPC (17.9) among anthracnose treatments, reduced disease progression during cold storage by approximately 64.3% and, *in vivo*, delayed ripening by maintaining higher pulp firmness (7.30 N *versus* 3.42 N), sustaining a higher DA index (1.91 *versus* 1.77) and postponing the respiratory peak by 7 days at 9 °C.

The observed coating performance is consistent with a synergistic effect of CS, ZnO‐NPs and EEO, combining a physical barrier with bioactive mechanisms. From a sustainability perspective, the aqueous, acidified CS matrix and a plant‐derived essential oil support an approach that may reduce reliance on synthetic fungicides and is aligned with environmentally conscious postharvest practices.

Regarding practical implementation, the coating step can be integrated after fruit sanitisation and before packing, and is compatible with cold‐chain logistics at 9 °C. Future work should address scale‐up, for example dipping or spraying, process economics and throughput. Potential limitations include the need for regulatory clearance for nanoparticle use in edible coatings and an assessment of cost‐effectiveness at a commercial scale. Beyond mangoes, validation for other climacteric tropical commodities, such as papaya and banana, will help define the broader applicability of this multicomponent coating.

Taken together, the formulation enhanced microbial control and preserved postharvest quality of mango during cold storage, indicating potential to support longer distribution windows and maintain commercial quality, while reducing dependence on conventional chemical treatments. These outcomes underscore the relevance of the developed coating to the fruit industry and motivate pilot to commercial scale trials under diverse storage and transport scenarios.

## AUTHOR CONTRIBUTIONS

AGP: Conceptualisation, data curation, formal analysis, investigation, methodology, validation, writing – original draft. ACS: Data curation, investigation. HSGS: Data curation, investigation. DSR: Data curation, investigation, validation. MMC: Data curation, investigation, validation. BPCP: Data curation, investigation. PMRJ: Data curation, formal analysis, investigation, software, validation. STF: Data curation, formal analysis, investigation, validation, writing – original draft. DFMN: Conceptualisation, data curation, formal analysis, investigation, methodology, project administration, resources, supervision, validation, writing – original draft, writing – review and editing.

## FUNDING INFORMATION

This study was financed in part by the Coordenação de Aperfeiçoamento de Pessoal de Nível Superior‐Brasil (CAPES)‐Finance Code 001, Conselho Nacional de Desenvolvimento Científico e Tecnológico (CNPq) and Fundação de Amparo à Ciência e Tecnologia do Estado de Pernambuco (FACEPE) (IBPG‐1650‐2.00/21).

## CONFLICT OF INTEREST

The authors declare that they have no conflicts of interest.

## Data Availability

The data that support the findings of this study are available from the corresponding author upon reasonable request.
